# Factors Associated with Late Antiretroviral Therapy Initiation among Adults in Mozambique

**DOI:** 10.1371/journal.pone.0037125

**Published:** 2012-05-15

**Authors:** Maria Lahuerta, Josue Lima, Harriet Nuwagaba-Biribonwoha, Mie Okamura, Maria Fernanda Alvim, Rufino Fernandes, Americo Assan, David Hoos, Batya Elul, Wafaa M. El-Sadr, Denis Nash

**Affiliations:** 1 Mailman School of Public Health, ICAP-Columbia University, New York, New York, United States of America; 2 Mailman School of Public Health, ICAP-Columbia University, Maputo, Mozambique; 3 Ministry of Health Advisor, Maputo, Mozambique; 4 Department of Epidemiology, Columbia University, Mailman School of Public Health, New York, New York, United States of America; Rollins School of Public Health, Emory University, United States of America

## Abstract

**Background:**

Despite recent changes to expand the ART eligibility criteria in sub-Saharan Africa, many patients still initiate ART in the advanced stages of HIV infection, which contributes to increased early mortality rates, poor patient outcomes, and onward transmission.

**Methods:**

To evaluate individual and clinic-level factors associated with late ART initiation in Mozambique, we conducted a retrospective sex-specific analysis of data from 36,411 adult patients who started ART between January 2005 and June 2009 at 25 HIV clinics in Mozambique. Late ART initiation was defined as CD4 count<100 cells/µL or WHO stage IV. Mixed effects models were used to identify patient- and clinic-level factors associated with late ART initiation.

**Results:**

The proportion of patients initiating ART late decreased from 46% to 37% during 2005–2007, but remained constant (between 37–33%) from 2007–2009. Of those who initiated ART late (median CD4 = 57 cells/µL), 5% were known to have died and 54% were lost to clinic within 6 months of ART initiation (compared with 2% and 47% among other patients starting ART [median CD4 = 192 cells/µL]). In multivariate analysis, female sex and pregnancy at ART initiation (AOR_female_not_pregnant_vs._male_ = 0.66, 95%CI [0.62–0.69]; AOR_pregnant_vs._non_pregnant_ = 0.60, 95%CI [0.49–0.73]), younger and older age (AOR_15–25_vs.26–30_ = 0.86, 95%CI [0.79–0.94], AOR_>45_vs.26–30_ = 0.72, 95%CI [0.67–0.77]), entry into care via PMTCT (AOR_entry_through_PMTCT_vs.VCT_ = 0.42, 95%CI [0.35–0.50]), marital status (AOR_married/in union_vs.single_ = 0.87, 95%CI [0.83–0.92]), education (AOR_secondary_or_higher_vs.primary_ = 0.87, 95%CI [0.83–0.93]) and year of ART initiation were associated with a lower likelihood of late ART initiation. Clinic-level factors independently associated with a lower likelihood of late ART initiation included CD4 machine on-site (AOR_CD4_machine_onsite_vs.offsite_ = 0.83, 95%CI [0.74–0.94]) and presence of PMTCT services onsite (AOR = 0.85, 95%CI [0.77–0.93]).

**Conclusion:**

The risk of starting ART late remained persistently high. Efforts are needed to ensure identification and enrollment of patients at earlier stages of HIV disease. Individual and clinic level factors identified may provide clues for upstream structural interventions.

## Introduction

In 2009, Sub-Saharan Africa was home to 68% of the estimated 33.3 million people living with HIV/AIDS worldwide [1]. Since 2004, there has been a rapid increase in antiretroviral therapy (ART) coverage in this region and approximately 37% of HIV-infected patients in need of treatment in sub-Saharan Africa had access to it in 2009 based on the criteria in the 2006 WHO guidelines [1]. Among the most important challenges to maximizing the impact of HIV care and treatment program outcomes are high rates of late ART initiation (i.e., initiation of ART in the advanced stages of HIV disease) [2]–[5], which in turn is associated with both high rates of mortality soon after initiation of ART (early mortality) and onward HIV transmission [6], [7].

Even though mortality in HIV-infected patients has decreased substantially with the scale-up of ART [6], mortality of patients starting ART in resource-limited settings has been shown to be substantially higher than in industrialized countries, especially in the first year following ART initiation [8]. In sub-Saharan Africa, one review article reported between 8 and 26% of patients die within 12 months after starting ART, with most deaths occurring in the first few months [6]. This high early mortality rate seems to be related in part to late ART initiation [9]. In fact, data from ART programs in 12 sub-Saharan countries found that most patients continue to start ART with CD4 counts well below the recommended threshold [10]. A cross-sectional study from a clinic in rural Uganda observed that 40% of the 2,311 patients initiating ART had WHO stage IV and found that male sex, lower education level and unemployment, among other factors, were associated with a higher likelihood of late ART initiation [11]. Identifying those factors associated with late ART initiation is critical to inform strategies to address this issue.

With an estimated national HIV prevalence of 11.5% in 2009 [12], Mozambique has experienced a rapid scale-up of HIV care and treatment programs, with the number of people on ART increasing from less than 2,000 in 2003 to more than 190,000 by July 2010 [13], [14]. Despite this accomplishment, it is estimated that only 37% of those in need of ART in Mozambique have initiated treatment [15]. A retrospective study looking at follow-up outcomes of 154,188 patients enrolled in 28 clinics between 2003 and 2009 in Mozambique observed low CD4 at ART initiation and high mortality rates [16]. Additionally, a study conducted in Zambezia province found that among those ART eligible at enrollment (21%), only 58% initiated ART within 90 days of enrollment, and older age and higher level of education were strongly predictive of ART initiation [17]. The objective of this analysis is to determine the extent of late ART initiation among adults across a large number of HIV care and treatment programs in Mozambique and identify patient and clinic-level factors associated with late ART initiation to help inform the development of upstream structural intervention.

## Methods

### Ethics Statement

This study was part of the Identifying Optimal Models of HIV Care and Treatment in Mozambique Collaboration and Multilevel Determinants of Late ART Initiation (LSTART) Study, which was approved by the Mozambican National Ethics Committee and the Columbia University Medical Center IRB. Additional technical and administrative approval was received from the US Centers for Disease Control and Prevention and the Office of the Global AIDS Coordinator (OGAC), US Department of State. Patients were not asked for informed consent to participate given that this study is solely based on secondary data analysis from de-identified routine service delivery data. Both as part of LSTART and the Optimal Models Collaboration, the Columbia University IRB Committee did not consider this study to be human subjects research as there was no interaction with subjects, there was no intervention and private, identifiable information was not being collected.

### Study population and ART eligibility

**Figure 1:**
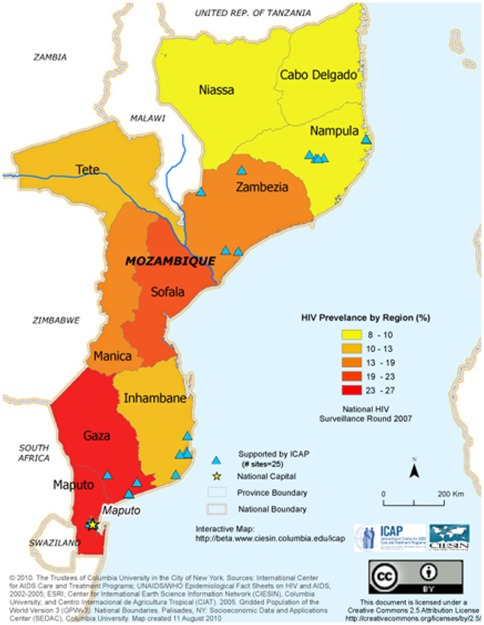
Geographic distribution of the 25 sites included in this analysis.

The study population included patients aged ≥15 who started ART from January 1, 2005 through June 30, 2009 at 25 HIV care and treatment clinics. All clinics were part of the national HIV care and treatment program in Mozambique and were located in urban and rural locations within five different provinces: Maputo, Gaza, Inhambane, Nampula and Zambezia (Figure 1). These HIV clinics were receiving direct support from ICAP- Columbia University through funding via PEPFAR.

During the study period, the Mozambican national guidelines (2006 & 2008) specified the following eligibility criteria for ART initiation: 1) WHO stage IV disease, 2) WHO stage III and CD4 count <350 cell/µL or 3) CD4 count <200 cells/µL, irrespective of WHO stage [18]. Since 2006, Mozambican guidelines further specified that all HIV-infected patients should have a comprehensive medical history taken, receive a physical examination and an appropriate laboratory assessment at their first HIV care visit to determine, among other things, ART eligibility. For patients who were not yet ART eligible, the guidelines specified a semiannual visit schedule with clinical and CD4+ cell count monitoring. Patients found to be ART eligible are required to attend three ART readiness counseling sessions and disclose their HIV status to a treatment partner (ie, parent, spouse, relative, friend). After this, the patient could be re-evaluated to confirm ART eligibility and readiness prior to prescription of ART. For patients with CD4<350 cells/µL or clinical WHO stage 3 or 4, cotrimoxazole was indicated.

### Outcome definition

Among those who initiated ART during the study period, patients for whom WHO staging or CD4 cell count values were available are included in the analysis. Late ART initiation was defined as having a CD4 count<100 cells/µL or WHO stage IV at any time prior to ART initiation or up to one month after ART initiation.

### Patient characteristics

Patient information routinely collected during each clinic visit was documented by clinicians on national patient forms included in patient charts. Trained data clerks routinely abstracted relevant data for baseline and all follow-up clinic visits from patient charts into an electronic database including: sex, age, weight, and WHO disease stages and CD4+ cell counts. Other variables included: pregnancy status at ART initiation, age, marital status, years of schooling, socio-economic status), source of referral (e.g., voluntary counseling and testing [VCT] service, TB clinic, prevention of mother-to-child transmission [PMTCT] program) and year of ART initiation. Loss to follow-up (LTF) was defined as not having had a clinic or pharmacy visit during the last 6 months of the study period (ie, Jan–Jun 2009).

### Clinic characteristics

Information on clinic and program characteristics was derived from annual structured site assessments completed by ICAP field staff at each clinic. These assessments captured information regarding: the type of setting (urban, rural), the type of facility (primary, secondary, tertiary), year the facility began providing ART, laboratory services (e.g., availability of CD4 testing), presence of patient support services (e.g. peer educators or outreach for patients who miss clinic visits) and presence of programmatic services (e.g. prevention of mother to child transmission program). Rounds of clinic assessments were conducted in June 2007, December 2007, July 2008 and July 2009, and linked to data on patients initiating ART during these time periods. Data from the June 2007 site assessment was used as a proxy for clinic characteristics prior to 2007.

### Statistical Analysis

Statistical analyses were carried out using SAS version 9.2 (SAS Institute, Cary, NC). Mixed effects models were used to identify patient- and clinic-level factors associated with late ART initiation using the GLIMMIX procedure in SAS. Variables significant at the 0.20 level in the univariate analysis were included in the multivariate analysis and, using a backward stepwise procedure, retained if significant at the 0.05 level based on the likelihood ratio test. Because the factors associated with late ART initiation likely differ by sex, we constructed sex-specific models. Sensitivity analyses were conducted to evaluate the impact of missing data in the final results.

## Results

### Study population

Between January 2005 and June 2009, 40,995 adult patients started ART at the 25 clinics, which represented an estimated 31.0% of all patients that had ever started ART in Mozambique by June 2009 [19]. Of those, 4,584 (11%) patients did not have a WHO stage or CD4 cell count to be classified as late or non-late ART initiators and were excluded from the main analysis (but examined under various assumptions in sensitivity analyses). The proportion of patients with missing WHO stage and CD4 count was greatest in 2005 (22%) but stabilized around 10% in the following years. Of the remaining 36,411 patients included in the main analysis, 27,784 patients (76%) had CD4 cell counts available at ART initiation and their median CD4 cell count was 150 cells/µL [interquartile range (IQR) 72–219] and 30,675 patients had WHO stage available at ART initiation (15% with WHO stage IV). A total of 14,010 (38%) of the 36,411 included in main analysis initiated ART late (WHO stage 4 and/or CD4 count <100 cells/µL), including 44% of men, 36% of non-pregnant women and 17% of pregnant women. Of the 14,010 patients who initiated ART late, 76% had a CD4 count<100 cells/µL, 34% had a WHO stage 4 and 10% had both at ART initiation.

### Patient characteristics at ART initiation and outcomes

Characteristics of the patients included are presented in Table 1. More than half were women (60%) and 852/16,361 (3.8%) were documented as being pregnant at ART initiation. The median age at ART initiation was 35.0 years (IQR 28.6–43.0), the median for women was 33.0 years (IQR 27.2–40.4) and for men 38.0 years (IQR 31.1–45.4). Among those with information on marital status, 56% of the patients were married or in union. Despite statistically significant differences in the distribution of patient-level characteristics between late and non-late initiators, only gender and calendar year of ART initiation had substantial differences in the distribution between both groups. Among those that initiated ART late (median CD4 = 57 cells/µL [IQR,25–89]), 5% were known to have died within 6 months and 54% were LTF; while among other patients initiating ART it was 2% and 47%, respectively (median CD4 = 192 cells/µL [IQR,151–255]). The proportion initiating ART late remained relatively constant (between 37–33%) from 2007 through the first half of 2009.

**Table 1:** Patient-level characteristics at ART initiation.^a^

Characteristics	Total		Late ART initiators		Non-late ART initiators		
	(N = 36,411)		(N = 14,010)		(N = 22,401)		p-value
	N	(%)	n	(%)	n	(%)	
**Sex**							
Male	13,731	37.7	6,117	43.7	7,614	34.0	<0.001
Female (not pregnant at ART initiation)	21,828	59.9	7,752	55.3	14,076	62.8	
Female (pregnant at ART initiation)	852	2.3	141	1.0	711	3.2	
**Age (years)**							
15–25	3,920	10.8	1,322	9.4	2,598	11.6	<0.001
26–30	6,871	18.9	2,693	19.2	4,178	18.7	
31–35	7,452	20.5	2,951	21.1	4,501	20.1	
35–40	5,983	16.4	2,450	17.5	3,533	15.8	
41–45	4,755	13.1	1,930	13.8	2,825	12.6	
>45	7,430	20.4	2,664	19.0	4,766	21.3	
**Point of entry**							
VCT	12,077	33.2	4,650	33.2	7,427	33.2	<0.001
PMTCT	1,308	3.6	202	1.4	1,106	4.9	
TB/HIV	308	0.8	129	0.9	179	0.8	
Inpatient	1,802	4.9	868	6.2	934	4.2	
Outpatients	2,066	5.7	828	5.9	1,238	5.5	
Other^b^	16,015	44.0	6,118	43.7	9,897	44.2	
Missing	2,835	7.8	1,215	8.7	1,620	7.2	
**Marital status**							
Single	10,789	29.6	4,299	30.7	6,490	29.0	<0.001
Married/In union	17,181	47.2	6,397	45.7	10,784	48.1	
Widowed	2,913	8.0	1,021	7.3	1,892	8.4	
Missing	5,528	15.2	2,293	16.4	3,235	14.4	
**Years of schooling**							
None/very low (≤3 years)	1,733	4.8	676	4.8	1,057	4.7	0.009
Primary school (4–8 years)	17,603	48.3	6,737	48.1	10,866	48.5	
Secondary school or higher (>8 years)	8,216	22.6	3,069	21.9	5,147	23.0	
Missing	8,859	24.3	3,528	25.2	5,331	23.8	
**Socio-economic status**							
Higher	12,020	33.0	4,623	33.0	7,397	33.0	0.041
Lower	22,651	62.2	8,668	61.9	13,983	62.4	
Missing	1,740	4.8	719	5.1	1,021	4.6	
**Calendar year of ART initiation**							
2005	2,970	8.2	1,376	9.8	1,594	7.1	<0.001
2006	7,132	19.6	3,186	22.7	3,946	17.6	
2007	10,579	29.1	3,953	28.2	6,626	29.6	
2008	10,172	27.9	3,645	26.0	6,527	29.1	
First half 2009	5,558	15.3	1,850	13.2	3,708	16.6	

a Includes only individuals with available data.

b Includes referrals from other health facilities, youth centers, private clinics, laboratory, and emergency room.

### Clinic characteristics

Of the 25 clinics included in the analysis, 21 (84%) were located in urban settings and 4 (16%) were located in rural settings, with 7.8% of the patients coming from rural clinics (Table 2). All were public sector facilities with 11 primary facilities, 9 secondary and 5 tertiary facilities. CD4+ cell count testing was available onsite in 14(56%) of the facilities, with these clinics contributing the majority (71%) of the patients included in this analysis. Nineteen clinics (76%) reported having an outreach program for patients who missed clinic visits, seventeen of which were dedicated only for ART patients, while only two focused on all patients (pre-ART or ART) who missed visits. Finally, 21% (84%) had peer educators available and 21 (84%) had PMTCT services available on-site.

**Table 2:** Clinic and program characteristics of sites where patients started ART stratified by sex.

	Total no. sites		Total		Late ART initiators		Non-late ART initiators		
	(N = 25)		(N = 36,411)		(N = 14,010)		(N = 22,401)		p-value
	n (%)		N	(%)	n	(%)	n	(%)	
**Setting**									
Urban	21	84.0	33,401	91.7	13,208	94.3	20,437	91.2	<0.001
Rural	4	16.0	3,010	8.3	802	5.7	1,964	8.8	
**Type of facility**									
Primary	11	44.0	7,418	20.4	2,607	18.6	4,811	21.5	<0.001
Secondary	9	36.0	19,016	52.2	7,255	51.8	11,761	52.5	
Tertiary	5	20.0	9,977	27.4	4,148	29.6	5,829	26.0	
**Year the facility began providing ART**									
Before 2005	5	20.0	11,607	31.9	4,864	34.7	6,743	30.1	<0.001
2005	7	28.0	11,770	32.3	4,364	31.1	7,406	33.1	
2006	10	40.0	10,591	29.1	4,001	28.6	6,590	29.4	
2007	2	8.0	1,629	4.5	553	3.9	1,076	4.8	
2008	1	4.0	814	2.2	228	1.6	586	2.6	
**CD4 testing**									
On-site	14	56.0	26,160	71.8	10,252	73.2	15,908	71.0	<0.001
Off-site	11	44.0	10,251	28.2	3,758	26.8	6,493	29.0	
**Outreach program targeted to**									
All patients	2	8.0	11,500	31.6	4,584	32.7	6,916	30.9	<0.001
Only ART patients	17	68.0	14,021	38.5	5,199	37.1	8,822	39.4	
None	6	24.0	10,890	29.9	4,227	30.2	6,663	29.7	
**Peer education service**									
Yes	21	84.0	17,791	48.9	6,533	46.6	11,258	50.3	<0.001
No	4	16.0	18,620	51.1	7,477	53.4	11,143	49.7	
**Availability PMTCT services**									
On-site	21	84.0	23,587	64.8	8,726	62.3	14,861	66.3	<0.001
Off-site	4	16.0	12,824	35.2	5,284	37.7	7,540	33.7	

### Factors associated with late ART initiation

The results of the univariate and multivariate analyses for all patients, females, and males are shown in Appendix S1 and Table 3, respectively. In the multivariate analysis, non-pregnant women were significantly less likely than men to initiate ART late (AOR = 0.66, 95%CI [0.62–0.69]) as were pregnant women (AOR = 0.60, 95%CI [0.49–0.73]) when compared to non-pregnant women. Older patients (>45 years old) in the overall group had lower odds of late ART initiation (AOR = 0.72, 95%CI [0.67–0.77]), as did older women (AOR = 0.74, 95%CI [0.68–0.82]) and older men (AOR = 0.66, 95%CI [0.59–0.74]). Overall patients that entered care through inpatient settings had higher odds of late ART initiation than those entering through VCT (AOR = 1.4, 95%CI [1.3–1.6]). Among women, those entering care through PMTCT services were significantly less likely to initiate ART late than those entering via VCT (AOR = 0.42, 95%CI [0.35–0.50]). Patients that were married or in union and those widowed had lower odds of late ART initiation compared to those that were single in the overall group (AOR = 0.87, 95%CI [0.83–0.92], AOR = 0.87,95% CI [0.83–0.92], respectively) and among women (married/in union AOR = 0.79,95%CI[0.74–0.85], widowed AOR = 0.85,95%CI[0.77–0.94]). Compared with having less education, having a secondary education or higher was associated with a lower odds of late ART initiation among all patients (AOR = 0.87, 95%CI [0.83–0.93]), and among men and women separately. Compared to those patients starting ART in 2005, those who initiated in more recent calendar years had lower odds of being late ART initiators but it stabilized after 2007.

**Table 3:** Multivariate analysis on factors associated with late ART initiation (CD4 count <100 cells/µL or WHO stage IV) stratified by patient gender and adjusting for clustering.^a^

Characteristics	Overall AOR		Women AOR		Men AOR	
	(95%CI)		(95%CI)		(95%CI)	
	(N = 36,411)		(N = 22,680)		(N = 13,731)	
*Patient-level characteristics*						
**Sex**						
Male	1					
Female (not pregnant at ART initiation)	**0.66**	**(0.62–0.69)**	1		-	-
Female (pregnant at ART initiation)	**0.38**	**(0.31–0.47)**	**0.60**	**(0.49–0.73)**	-	-
**Age (years)**						
15–25	**0.86**	**(0.79–0.94)**	**0.90**	**(0.81–0.99)**	**0.72**	**(0.61–0.87)**
26–30	1		1		1	
31–35	0.96	(0.89–1.0)	0.96	(0.89–1.0)	0.93	(0.83–1.0)
35–40	0.97	(0.90–1.0)	0.96	(0.88–1.1)	0.94	(0.84–1.1)
41–45	**0.91**	**(0.84–0.99)**	**0.88**	**(0.79–0.97)**	**0.92**	**(0.81–1.0)**
>45	**0.72**	**(0.67–0.77)**	**0.74**	**(0.68–0.82)**	**0.66**	**(0.59–0.74)**
**Point of entry**						
VCT	1		1		1	
PMTCT	**0.42**	**(0.35–0.50)**	**0.42**	**(0.35–0.50)**	0.69	(0.36–1.3)
TB/HIV	1.2	(0.98–1.6)	**1.4**	**(1.0–2.0)**	1.1	(0.79–1.5)
Inpatient	**1.4**	**(1.3–1.6)**	**1.5**	**(1.3–1.7)**	**1.3**	**(1.1–1.5)**
Outpatients	0.97	(0.86–1.1)	1.1	(0.94–1.3)	**0.84**	**(0.70–0.99)**
Other^b^	1.0	(0.86–1.1)	1.0	(0.94–1.3)	**0.88**	**(0.79–0.97)**
Missing	1.1	(0.97–1.2)	1.1	(0.99–1.3)	1.0	(0.83–1.1)
**Marital status**						
Single	1		1		-	-
Married/In union	**0.87**	**(0.83–0.92)**	**0.79**	**(0.74–0.85)**	-	-
Widowed	**0.87**	**(0.83–0.92)**	**0.85**	**(0.77–0.94)**	-	-
Missing	0.92	(0.84–1.0)	0.93	(0.83–1.0)	-	-
**Years of schooling**						
None/very low (≤3 years)	1.1	(0.99–1.2)	1.1	(0.93–1.2)	1.2	(0.99–1.6)
Primary school (4–8 years)	1		1		1	
Secondary school or higher (>8 years)	**0.87**	**(0.83–0.93)**	**0.85**	**(0.79–0.92)**	**0.89**	**(0.82–0.97)**
Missing	1.0	(0.95–1.1)	1.0	(0.93–1.1)	1.0	(0.92–1.1)
**Calendar year of ART initiation**						
2005	1		1		1	
2006	**0.95**	**(0.87–1.0)**	0.91	(0.81–1.0)	**1.0**	**(0.88–1.2)**
2007	**0.71**	**(0.65–0.78)**	**0.66**	**(0.59–0.74)**	**0.79**	**(0.69–0.90)**
2008	**0.70**	**(0.64–0.76)**	**0.64**	**(0.57–0.72)**	**0.79**	**(0.68–0.90)**
First half 2009	**0.69**	**(0.62–0.76)**	**0.64**	**(0.56–0.74)**	**0.74**	**(0.63–0.87)**
*Program characteristics*						
**Setting**						
Urban	-	-	-	-	1	
Rural	**-**	**-**	-	-	**0.73**	**(0.54–0.99)**
**CD4 testing**						
On-site	**0.83**	**(0.74–0.94)**	**0.86**	**(0.75–0.98)**	**0.83**	**(0.54–0.99)**
Off-site	1		1		1	-
**Availability PMTCT services**						
On-site	**0.85**	**(0.77–0.93)**	**0.81**	**(0.72–0.91)**	-	-
Off-site	1		1		-	-

CI, confidence interval.

a Includes only individuals with available data.

b Includes referrals from other health facilities, youth centers, private clinics, laboratory, and emergency room.

Clinic-level factors that were independently associated with late ART initiation among all patients included availability of CD4 cell count testing onsite versus offsite (AOR = 0.83, 95%CI [0.74–0.94]) and onsite versus offsite availability of PMTCT services (AOR = 0.85, 95%CI [0.77–0.93]). In the multivariate model for women, onsite availability of CD4 cell count testing (AOR = 0.86, 95%CI [0.75–0.98]) and PMTCT services (AOR = 0.81, 95%CI [0.72–0.91]) were significantly associated with lower odds of late ART initiation. Finally, men initiating ART at rural clinics (AOR_rural_vs._urban_ = 0.73, 95%CI [0.54–0.99]) and clinics with onsite CD4 cell count testing (AOR = 0.83, 95%CI [0.54–0.99]) were significantly less likely to initiate ART late. Sites with peer education programs were significantly associated with a lower likelihood of late ART initiation in univariate analysis (Table 3). However, peer educator programs were no longer significant after controlling for other patient and program-level factors, and having a more recent year of ART initiation appeared to explain most of the relationship between peer education and late ART initiation.

### Sensitivity analysis

In order to evaluate the impact of missing data on the outcome of late ART initiation on our main findings, sensitivity analyses were conducted with the 4,584 patients that had been excluded from the main analysis due to missing information on WHO stage and/or CD4 cell count at ART initiation. The proportion of patients with late ART initiation among all patients who initiate ART ranged from 34%, if those with missing WHO stage or CD4 cell count at ART initiation are assumed to not have initiated ART late, to 45%, if all those with these missing data are classified as having initiated ART late. Excluded patients were incorporated in multivariate model and treated as late ART initiators in one model and as non-late ART initiators in a second model. When we compared these models to that in Table 3, the findings were not materially altered.

## Discussion

Our study found a median CD4 cell count at ART initiation of 150 cells/µL among patients who initiated ART at 25 HIV care and treatment clinics in Mozambique. A high proportion of patients (38%, range across sites: 21%–52%) started ART late (CD4<100 or WHO IV) and of these, 5% were known to have died and 54% were lost to clinic within 6 months of ART initiation. These data are consistent with several other studies in the sub-Saharan African region [3], [4], [6], [8]–[11], [16], [20]–[29]. Our study identified several individual- and clinic-level factors that were independently associated with late ART initiation, some of which are modifiable and can thus be utilized to inform upstream, structural interventions aimed at promoting more timely ART initiation.

A number of individual-level factors were found to be independently associated with a lower likelihood of late ART initiation, including female sex, pregnancy at ART initiation, entry into care via a PMTCT program, younger (15–25 years) and older age (>40 years), more years of education and being married or widowed (among females only). In sub-Saharan Africa, women on average initiate ART at an earlier stage of HIV-disease than men [28], [30]. One likely reason is the expansion of HIV testing for pregnant women in antenatal care through PMTCT programs leading to identification of HIV among asymptomatic women [31], [32]. Some approaches to reach men earlier include promotion of HIV testing in general among populations with high HIV prevalence, support to workplace HIV testing programs [33], [34] as well as to promote HIV testing among household members of HIV-infected individuals [35]–[37]. In fact, even though pediatric children were not the included in this analysis, late HIV diagnosis in adults in Mozambique are a serious problem in the late diagnosis of infants with HIV as well [38]. The observed association with younger age and lower risk of late ART initiation seems plausible since younger age is likely associated with both a shorter duration of HIV infection and higher testing rates in most sub-Saharan African countries [39]–[44]. The association with older age and lower risk of late ART initiation is consistent with what has recently been described in rural Mozambique [17]. Among women, being widowed or married/in union was associated with lower risk of late ART initiation when compared to those who were single. The death of a spouse could prompt an individual to seek an HIV test prior to the onset of symptoms, especially if HIV is suspected as a possible cause death in the spouse, and further motivate an individual to seek HIV care for already known HIV infection. There are other factors that we were not able to assess that have been previously described as barriers for retention in care and early ART initiation, including distance and cost of transportation to clinic, work responsibilities, family commitments or cumbersome ART initiation procedures [17], [39].

The risk of late ART initiation between 2005 and 2007 decreased from 46% to 37%. This may reflect the effect of rapidly increasing ART coverage [40], more efficient triaging of the sickest patients, and increasing HIV testing uptake among HIV-infected persons in Mozambique. However, the proportion of patients initiating late in our sample changed only slightly from 2007 through the first half of 2009. This leveling off is concerning and community and clinic level interventions that focus on earlier diagnosis and enrollment into care are urgently needed [5]. In sub-Saharan Africa, HIV testing uptake is variable [41]–[52], but generally low throughout the region (e.g., in 2008 only 22% of people 15–49 years old knew their HIV status [40]), despite the availability of ART. As testing and ART coverage increase, rates of late ART initiation would be expected to further decrease. However, the plateau in our data suggests a suboptimal steady state may have been reached in the catchment areas of these 25 clinics.

A number of clinic-level factors were found to be independently associated with late ART initiation. Male patients enrolled at rural clinics and clinics were less likely to initiate ART late (consisting with a prior investigation [4], although men in rural sites represented only 8% of the men included in this analysis. Availability of CD4+ cell count testing onsite (as opposed to offsite) was associated with a lower risk of late ART initiation in both females and males. This might reflect ease of obtaining such tests and the ability of the clinicians to more closely monitor CD4+ cell count results and determine ART eligibility in a timely manner. It could also reflect other unmeasured factors related to the clinic maturity and capacity to provide a higher quality of pre-ART care. A substantial proportion of clinics in our analysis did not have CD4 testing available onsite (52%) and 29% of patients in this analysis were recruited at such clinics. The availability of cost-effective point of care CD4 analyzers could facilitate the use of such assays at smaller clinics enabling better monitoring of pre-ART patients and allowing for timely initiation of ART once eligible [53]. Women initiating ART at clinics that also had an on-site PMTCT program were significantly less likely to initiate ART late, independently of whether individual women entered the program via PMTCT. This may reflect ease of referral of HIV-infected women from PMTCT to HIV care and treatment services when both are co-located at the same facility.

Our study has several strengths including a sex-specific analysis and the use of routinely collected program data on a large number of patients from several clinics in diverse settings throughout Mozambique, each of which enhances the generalizability of our findings. The inclusion of patient as well as clinic characteristics in the analysis is also an added strength. However, our study also has several limitations. Measurement error on individual-level factors such as socio-economic status could have undermined our ability to observe true associations with late ART initiation, and resulted in inadequately controlled confounding by socio-economic status. We examined the role of clinic-level characteristics that were observed, rather than randomly assigned (i.e., experimentally), and as such observed associations may be prone to unmeasured confounding. We also lack information for time dependent clinic characteristics before 2007 and, instead used data from 2007 as a proxy. Finally, 11% of the patients had missing data on the parameters of interest for this analysis i.e. WHO staging and CD4+ cell count and thus were excluded from the main primary analysis. We assessed the impact of the missing data via sensitivity analyses with extreme assumptions and found that the findings were similar to those from the main analysis. However, this effort and the findings do not exclude the possibility that point estimates could be biased as a result of missing data.

Since 2009, many programmatic changes have occurred in Mozambique that could have contributed to reducing the problem of late ART initiation. Mozambique started decentralizing the health system in 2009, providing care in smaller health centers and expanding access to HIV care and treatment throughout the country. Also in 2009, national ART guidelines were changed to start patients with CD4 count <250 cells/mL independent of clinical status, CD4 count <350 cells/mL and WHO stage III, or WHO stage IV independent of CD4 count. More recently, point-of-care CD4 testing is currently being used in sites that previously did not have CD4 machines, with the potential reduction on the time from enrollment to ART initiation [54].

In conclusion, we observed that a large proportion of patients at sites included in this study initiated ART in the late stages of HIV disease. It is unclear the extent to which this is driven by distinct upstream factors along the pathway to ART initiation – late HIV diagnosis, late enrollment into HIV care, and late ART initiation despite timely enrollment in care. The decreasing trend in late ART initiation with calendar time since 2005 is encouraging but it seems to have plateaued since 2007. Thus, upstream, structural intervention strategies aimed at improving HIV testing coverage, linkage to HIV care following diagnosis, retention in pre-ART care with timely CD4 monitoring, and prompt ART initiation following eligibility are urgently needed [4], [5]. Some potential interventions to achieve this include the use of point-of-care CD4 testing, comprehensive service packages that integrate PMTCT services on site with ART programs and the support of community ART groups to improve access, patient retention, and decongest health services [39], [55].The associations with late ART initiation observed in our analyses require additional investigation, but may represent potentially modifiable determinants of late ART initiation in this setting. Furthermore, efforts to ensure timely ART initiation will need to be expanded with recent changes in WHO guidelines for ART use in resource limited settings and recent changes in the Mozambican National guidelines that will also expand eligibility to patients with higher CD4 cell counts [56]–[60].

## Supporting Information

Appendix S1 Univariate analysis table.
(DOCX)
