# Supplementary material for: Factors Associated with Late Antiretroviral Therapy Initiation among Adults in Mozambique
Source: PLoS One. 2012 May 15;7(5):e37125. doi: 10.1371/journal.pone.0037125 (PMC3352894; doi:10.1371/journal.pone.0037125)
Supplement: Appendix S1 — Univariate analysis table. (DOCX) [file pone.0037125.s001.docx]

**Appendix S1.** Univariate analysis on factors associated with late ART initiation (CD4 count <100 cells/µL or WHO stage IV) adjusting for clustering^a^

|  | | Overall OR | | Women OR | | Men OR | |
| --- | --- | --- | --- | --- | --- | --- | --- |
| Characteristics | (95%CI) | | | (95%CI) | | (95%CI) | |
|  | | (N=36,411) | | (N=22,680) | | (N=13,731) | |
| *Patient-level characteristics* | |  |  |  |  |  |  |
| **Sex** | |  |  |  |  |  |  |
| Male | | 1 |  |  |  |  |  |
| Female (not pregnant at ART initiation) | | **0.68** | **(0.65-0.71)** | 1 |  | - |  |
| Female (pregnant at ART initiation) | | **0.26** | **(0.21-0.31)** | **0.37** | **(0.30-0.45)** | - |  |
| **Age (years)** | |  |  |  |  |  |  |
| 15-25 | | **0.80** | **(0.74-0.87)** | **0.88** | **(0.80-0.97)** | **0.72** | **(0.61-0.86)** |
| 26-30 | | 1 | 1 | 1 |  | 1 | 1 |
| 31-35 | | 1.0 | (0.94-1.1) | 0.99 | (0.91-1.1) | 0.93 | (0.82-1.0) |
| 35-40 | | 1.1 | (0.98-1.1) | 1.0 | (0.93-1.1) | 0.94 | (0.84-1.1) |
| 41-45 | | 1.00 | (0.96-1.1) | 0.96 | (0.87-1.1) | 0.92 | (0.82-1.0) |
| >45 | | **0.84** | **(0.78-0.90)** | **0.82** | **(0.75-0.90)** | **0.67** | **(0.60-0.75)** |
| **Point of entry** | |  |  |  |  |  |  |
| VCT | | 1 |  | 1 |  | 1 |  |
| PMTCT | | **0.29** | **(0.25-0.34)** | **0.32** | **(0.27-0.38)** | 0.65 | (0.34-1.2) |
| TB/HIV | | 1.2 | (0.97-1.6) | 1.3 | (0.94-1.8) | 1.1 | (0.76-1.5) |
| Inpatient | | **1.4** | **(1.2-1.5)** | **1.4** | **(1.3-1.6)** | **1.2** | **(1.1-1.5)** |
| Outpatients | | 1.0 | (0.85-1.1) | 1.1 | (0.91-1.2) | **0.81** | **(0.68-0.96)** |
| Other^b^ | | **0.9** | **(0.86-0.97)** | 0.96 | (0.89-1.0) | **0.84** | **(0.76-0.92)** |
| Missing | | 1.1 | (1.0-1.2) | **1.2** | **(1.0-1.3)** | 0.97 | (0.84-1.1) |
| **Marital status** | |  |  |  |  |  |  |
| Single | | 1 |  | 1 |  | 1 | 1 |
| Married/In union | | **0.90** | **(0.86-0.95)** | **0.76** | **(0.71-0.81)** | 0.94 | (0.86-1.0) |
| Widowed | | **0.80** | **(0.74-0.87)** | **0.84** | **(0.76-0.92)** | 0.83 | (0.68-1.0) |
| Missing | | 1 | (0.95-1.1) | 1 | (0.92-1.1) | 0.97 | (0.85-1.1) |
| **Years of schooling** | |  |  |  |  |  |  |
| None/very low (≤3 years) | | 1 | (0.90-1.1) | 1.0 | (0.92-1.2) | 1.2 | (0.97-1.5) |
| Primary school (4-8 years) | | 1 |  | 1 |  | 1 |  |
| Secondary school or higher (>8 years) | | **0.92** | **(0.87-0.97)** | **0.86** | **(0.80-0.93)** | 0.91 | (0.84-1.0) |
| Missing | | 1 | (0.94-1.1) | 1 | (0.97-1.1) | 1.0 | (0.93-1.1) |
| **Socio-economic status** | |  |  |  |  |  |  |
| Higher | | 0.98 | (0.93-1.0) | 0.95 | (0.89-1.0) | 0.99 | (0.91-1.1) |
| Lower | | 1 |  | 1 |  | 1 |  |
| Missing | | 1.2 | (0.81-1.6) | 1.3 | (0.88-1.8) | 0.95 | (0.63-1.4) |
| **Calendar year of ART initiation** | |  |  |  |  |  |  |
| 2005 | | 1 |  | 1 |  | 1 |  |
| 2006 | | 0.96 | (0.88-1.0) | 0.93 | (0.83-1.0) | 1.02 | (0.89-1.2) |
| 2007 | | **0.71** | **(0.66-0.78)** | **0.67** | **(0.60-0.75)** | **0.79** | **(0.69-0.90)** |
| 2008 | | **0.68** | **(0.62-0.74)** | **0.63** | **(0.56-0.70)** | **0.76** | **(0.67-0.88)** |
| First half 2009 | | **0.63** | **(0.57-0.70)** | **0.59** | **(0.52-0.67)** | **0.70** | **(0.60-0.82)** |
| *Program characteristics* | |  |  |  |  |  |  |
| **Setting** | |  |  |  |  |  |  |
| Urban | | 1 |  | 1 |  | 1 |  |
| Rural | | **0.77** | **(0.62-0.96)** | 0.90 | (0.70-1.2) | **0.71** | **(0.54-0.95)** |
| **Type of facility** | |  |  |  |  |  |  |
| Primary | | 1 | 1 | 1 |  | 1 |  |
| Secondary | | 1.1 | (0.89-1.3) | 1.0 | (0.81-1.3) | 1.2 | (0.92-1.5) |
| Tertiary | | 1.2 | (0.87-1.6) | 1.1 | (0.78-1.5) | 1.2 | (0.88-1.7) |
| **Program size (# of patients on ART)** | |  |  |  |  |  |  |
| 1st quartile (<=783) | | 1.0 | (0.76-1.4) | 1.2 | (0.87-1.7) | 0.87 | (0.61-1.2) |
| 2nd quartile (784-1,021) | | 1.1 | (0.83-1.5) | 1.2 | (0.84-1.6) | 1.1 | (0.81-1.6) |
| 3rd quartile (1,022-2,290) | | 0.87 | (0.63-1.2) | 0.90 | (0.65-1.3) | 0.8 | (0.56-1.1) |
| 4th quartile (>2,290) | | 1 |  | 1 |  | 1 |  |
| **Year the facility began providing ART** | |  |  |  |  |  |  |
| Before 2005 | | 1.2 | (0.83-1.6) | 1.2 | (0.81-1.6) | 1.2 | (0.85-1.7) |
| 2005 | | 1 |  | 1 |  | 1 |  |
| 2006 | | 1.2 | (0.87-1.5) | 1.2 | (0.88-1.6) | 1.1 | (0.84-1.5) |
| 2007 | | 0.97 | (0.61-1.5) | 1.1 | (0.65-1.7) | 0.82 | (0.48-1.4) |
| 2008 | | 0.71 | (0.39-1.3) | 0.74 | (0.81-1.6) | 0.64 | (0.85-1.7) |
| **CD4 testing** | |  |  |  |  |  |  |
| On-site | | **0.77** | **(0.69-0.87)** | **0.82** | **(0.71-0.94)** | **0.79** | **(0.66-0.95)** |
| Off-site | | 1 |  | 1 |  | 1 |  |
| **Outreach program targeted to** | |  |  |  |  |  |  |
| All patients | | 1 | (0.95-1.1) | 0.99 | (0.90-1.1) | 1.1 | (0.94-1.2) |
| Only ART patients | | 0.97 | (0.91-1.0) | 0.98 | (0.90-1.1) | 0.94 | (0.85-1.0) |
| None | | 1 |  | 1 |  | 1 |  |
| **Peer education service** | |  |  |  |  |  |  |
| Yes | | **0.86** | **(0.81-0.92)** | **0.84** | **(0.77-0.92)** | 0.91 | (0.82-1.0) |
| No | | 1 |  | 1 |  | 1 |  |
| **Availability PMTCT services** | |  |  |  |  |  |  |
| On-site | | **0.81** | **(0.75-0.88)** | **0.78** | **(0.70-0.86)** | **0.87** | **(0.77-1.0)** |
| Off-site | | 1 |  | 1 |  | 1 |  |

CI, confidence interval;

^a^ Includes only individuals with available data

^b^ Includes referrals from other health facilities, youth centers, private clinics, laboratory, and emergency room
